# Angiosarcoma in an Arteriovenous Fistula Following Renal Transplantation: A Case Report and Literature Review

**DOI:** 10.7759/cureus.75611

**Published:** 2024-12-12

**Authors:** Charbel B Aoun, Elie Moukawam, Joseph Sfeir, Ziad Sleiman, George Ghanime

**Affiliations:** 1 Plastic and Reconstructive Surgery Department, Lebanese University Faculty of Medical Sciences, Beirut, LBN; 2 Plastic and Reconstructive Surgery Department, Lebanese Hospital Geitawi University Medical Center, Beirut, LBN; 3 Plastic and Maxillofacial Surgery Department, Al-Zahraa Hospital University Medical Center, Beirut, LBN; 4 Bioethics Department, Lebanese University Faculty of Medical Sciences, Beirut, LBN

**Keywords:** angiosarcoma, arteriovenous fistula, case report, diagnosis, hemodialysis vascular access, renal transplant

## Abstract

Angiosarcoma is a rare and aggressive malignant tumor arising from vascular or lymphatic endothelial cells. Angiosarcoma at an arteriovenous fistula site is exceptionally rare. We report a case of a 37-year-old male renal transplant recipient who developed a high-grade epithelioid angiosarcoma at the site of an arteriovenous fistula six years post-transplant. The lesion presented as a bleeding, enlarging mass and was initially misdiagnosed, delaying definitive treatment. A biopsy confirmed the diagnosis, and the patient underwent radical surgery, chemotherapy, and radiotherapy.

This case highlights the need for heightened clinical suspicion, particularly in patients with arteriovenous fistula-related complications, as early diagnosis and treatment are crucial for this rare but aggressive malignancy.

## Introduction

Cutaneous sarcomas are relatively uncommon and make up approximately 5% of all skin malignancies [1]. Among them, angiosarcomas are notably rare, accounting for less than 1% of all sarcomas [2-5]. Their prevalence is less than 1% of all malignant tumors and 2% of localized soft tissue cancers in kidney transplant recipients [6]. Angiosarcomas are malignant mesenchymal tumors that arise from lymphatic vessels or vascular endothelial cells and develop in different soft tissues and organs [2-5]. The skin (particularly the head and neck) is the most frequently affected area, followed by the breast, deep soft tissues, visceral organs, and bones [5,7,8]. The mean age of diagnosis was 73 years. Most angiosarcomas were found in White patients, and the tumor sizes ranged between 0.2 and 35 cm [9].

Angiosarcoma presents as a highly infiltrative tumor, prone to local destruction and metastasis, most often to the lungs and brain. Timely diagnosis is necessary due to the poor prognosis associated with the metastatic stage. The five-year survival rate is 45% for angiosarcoma patients, with overall survival time ranging from 6 to 16 months [10]. Numerous risk factors can lead to angiosarcoma, with arteriovenous fistulas (AVFs) being one of them [11]. However, reports of angiosarcoma at the site of an AVF are very rare.

To our knowledge, approximately 37 case reports in the literature have documented the development of angiosarcoma at an AVF site. We present a patient with an enlarging, bleeding tumor on his right arm located at the site of his AVF.

## Case presentation

**Figure 1:**
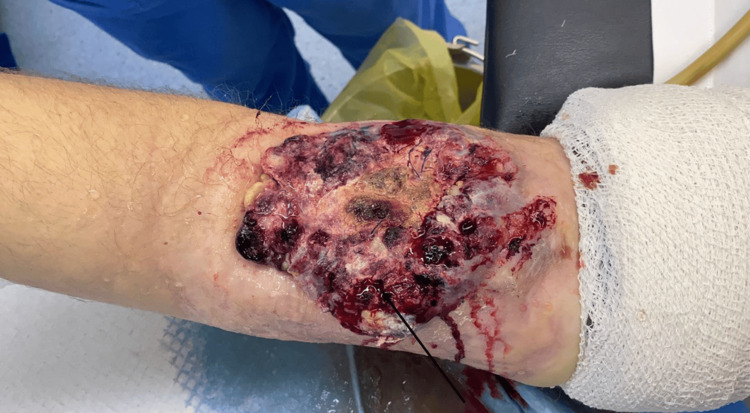
A large, ulcerated mass at the arteriovenous fistula, with bleeding vessels and necrosis (the black arrow indicates the ulcerated tumor).

A 37-year-old man with a functioning renal transplant presented to another facility with a nodular red mass over the brachio-cephalic AVF at his right arm that was done 13 years earlier due to end-stage renal disease. It served as the primary access site from 2011 to 2018. In 2018, the patient received a living donor renal transplant, and the AVF was closed five months later. He had been treated with immunosuppressive therapy consisting of mycophenolate mofetil (500 mg twice daily), prednisolone (5 mg once daily), and tacrolimus (3 mg once daily). That mass appeared six years after the kidney transplantation. The primary diagnosis was of a complicated thrombosed AVF that was treated by surgical excision of the thrombosed AVF, but no tissue was sent for histology. After the surgery, the patient complained of intractable bleeding from that area. Bleeding arterioles arising from the radial aspect of the brachial artery were identified by arteriography and embolized using several metallic micro coils until adequate stasis was achieved with remaining extremely small capillaries within the malformation, impossible to catheterize and treat by the endovascular route. One week later, he presented to our facility with a large, ulcerated mass at the AVF, with bleeding vessels, hematoma, and necrosis (Figure 1).

**Figure 2:**
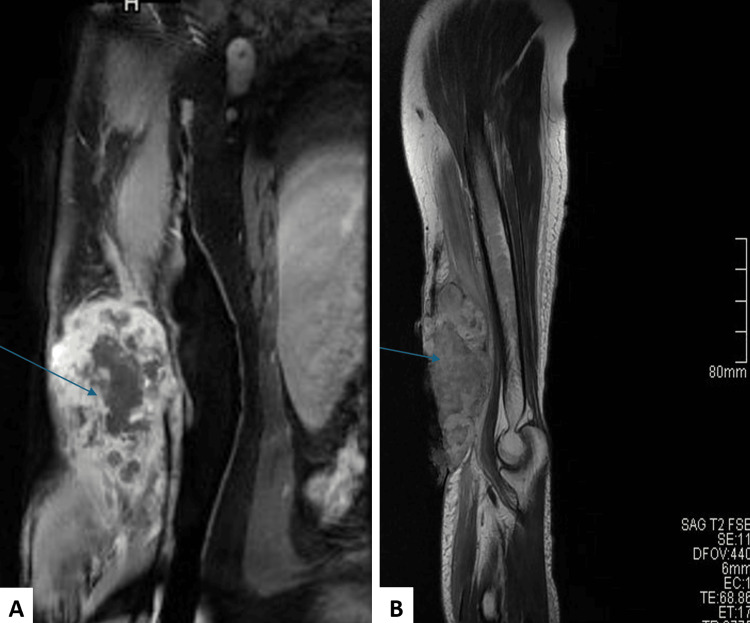
Imaging showing the coronal MRI of the right arm highlighting the heterogeneous, lobulated tumor mass (A) and the sagittal T2-weighted MRI demonstrating tumoral invasion and vascularization (B). Arrow in (A): the tumor is seen involving the anterior aspect of the distal right arm with skin ulceration and exposure of tumoral tissue. Arrow in (B): the tumor is shown invading the biceps brachialis, brachialis, and anteromedial brachioradialis with vascularization via the radial collateral artery.

He was anemic with a hemoglobin level of 6 g/dL (reference range: 13-17 g/dL). Compressive dressing was done, and MRI showed a heterogeneous lobulated mass in the anterior aspect of the distal right arm measuring 13 cm in craniocaudal dimension, 7.8 cm in transverse, and 5 cm in anteroposterior dimension. It was associated with a large ulcer, loss of overlying skin, and hypodermic fat; tumoral tissue was probably exposed. It involved the biceps brachialis, brachialis, and, focally, the anteromedial part of the superior aspect of the brachioradialis. It was vascularized via a rich network primarily from the radial collateral artery. The findings suggested an invasive, richly vascularized tumor with skin ulceration and an enlarged right axillary lymph node around the epitrochlear lymph node (Figure 2).

**Figure 3:**
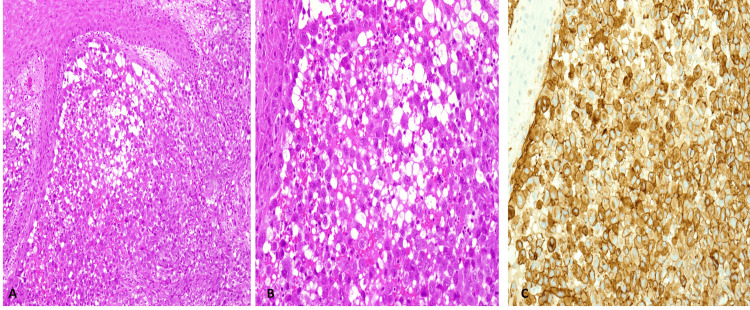
Histopathology results of the tumor. (A) Slide showing deep dermal and subdermal proliferation of atypical endothelial cells with anisonucleosis, large nuclei, and frequent mitoses. (B) Slide demonstrating frequent mitosis and prominent large nuclei. (C) Slide showing immunohistochemistry demonstrating CD31 positivity.

Biopsy of the lesion was taken in the operation room using a tourniquet to decrease blood loss and sent for pathology, which showed deep dermal and subdermal proliferation of atypical endothelial cells with large nuclei, frequent mitoses, and CD31 positivity on immunohistochemistry (Figure 3).

**Figure 4:**
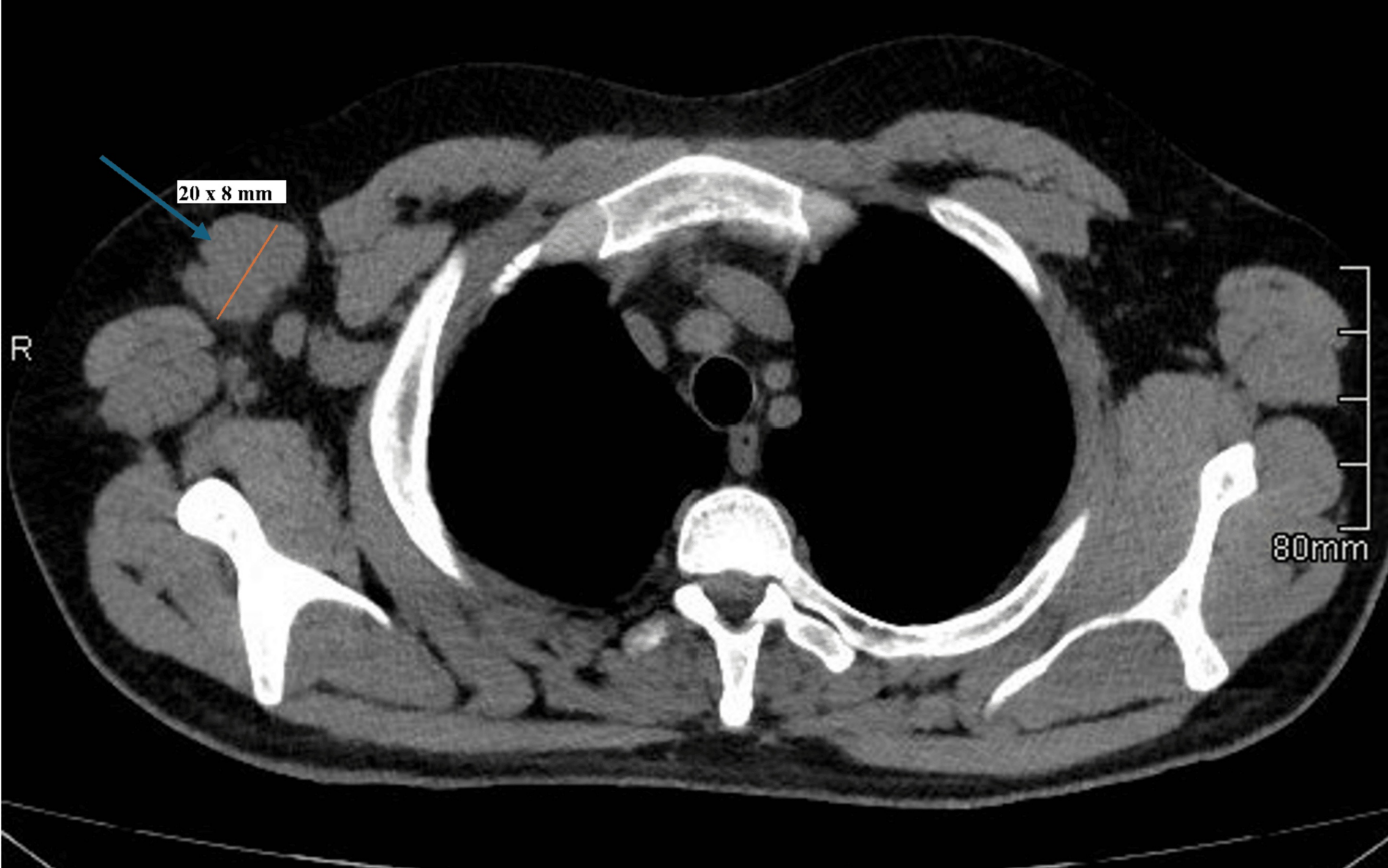
Axial CT scan of the chest showing the largest right axillary lymph node, measuring 20 x 8 mm (blue arrow).

These findings confirmed the diagnosis of high-grade angiosarcoma. Computed tomography (CT) of the thorax, abdomen, and pelvis showed multiple enlarged right axillary lymph nodes, with the largest measured at 20 x 8 mm (Figure 4).

After a multidisciplinary discussion, shoulder disarticulation was performed in another facility (due to intractable bleeding from the tumor and surrounding vessels, along with severe anemia caused by blood loss), followed by chemotherapy and radiotherapy (RT). To date (one year since the official diagnosis of angiosarcoma), the patient is alive and has completed both chemotherapy and RT sessions without complications, according to his own reports, although he has not maintained regular follow-up with our team.

## Discussion

The same search terms as in the reviews of Oskrochi et al. [12] and Van Acker et al. [13] were used: “angiosarcoma” and “arteriovenous fistula.” Systematic searches were conducted, and articles were extracted from Excerpta Medica Database (EMBASE), Online Database (OVID), and PubMed for the period spanning January 2020 to October 2024. Articles or cases describing angiosarcoma in relation to an AVF were included and those unrelated were excluded. Our search strategy returned 509 results. After applying the inclusion and exclusion criteria based on the title and abstract, three articles were accepted and deemed relevant for our review, which adds to the 22 cases already reviewed by Oskrochi et al. [12], and the 12 cases discussed by Van Acker et al. [13]. The three articles were case reports.

As of October 2024, to the best of our knowledge, 37 unique patients with angiosarcoma at an AVF site were mentioned in the literature, in addition to the present case (for a total of 38 cases). The data extracted from the case reports are summarized in Table 1. All three patients were male with epithelioid angiosarcoma. Two AVFs were thrombosed. All three cases had a functioning renal transplant. At the time of diagnosis, none of the AVFs were actively used. Two patients were still receiving immunosuppressive therapy because they had a functioning renal transplant. The third report did not specify whether the patient was on immunosuppressive therapy. One patient was on corticosteroids (prednisone), tacrolimus, and mycophenolate mofetil, and the second patient received tacrolimus. All cases reported pain at the site of the AVF as the initial symptom. Moreover, two patients had metastasis at presentation, and the third report did not state this information [14-16].

**Table 1:** Summary of data extracted from case reports. AVF: arteriovenous fistula; AS: angiosarcoma; M: male; NS: not stated; CT: computed tomography; MRI: magnetic resonance imaging; MMF: mycophenolate mofetil

Reference	Sex	Age (Years)	Presenting Symptom	Site	AVF Status	Transplantation Status and Type	Immunosuppression Status	Initial Diagnosis	Investigations	Histology	Time From AVF to AS	Time From Transplant to AS	Metastasis	Management	Survival
Harrell and Broehm (2020) [14]	M	67	Pain	Radiocephalic	Thrombosed	Functional (cadaveric kidney transplant)	NS	Thrombosed AVF	Excision histology	Epithelioid	NS	12	NS	Re-excision for complete margination of the lesion	NS
Kittitirapong et al. (2020) [15]	M	58	Pain	Right brachiocephalic	Thrombosed	Functional (living related kidney transplantation)	Prednisolone, tacrolimus, MMF	Thrombosed AVF, ruptured of an AVF aneurysm	Radiograph of the arm, chest computed tomography (CT), CT angiography, brachial arterial embolectomy for cytologic examination, tissue and bone biopsy for histology	Epithelioid	5	4	Lung, bone	Shoulder disarticulation, chemotherapy	1 month
D’Ambrosio et al. (2020) [16]	M	56	Pain and swelling mass	Left radiocephalic	Closed	Functional (cadaveric kidney transplant)	Tacrolimus	Complicated AVF	Radiogram, Doppler ultrasound and CT scan of the wrist, bone biopsy, total-body CT scan	Epithelioid	17	6	The lungs, the left iliac wing, and the duodenum	Left arm amputation (above elbow)	5 months
Present case, 2024	M	37	Swelling mass	Right brachiocephalic	Closed	Functional (living related kidney transplantation)	Prednisolone, tacrolimus, MMF	Complicated thrombosed AVF	Surgical revision of the AVF, arteriography, endovascular embolization, MRI, excisional biopsy and histology, total-body CT scan	Epithelioid	13	6	Right axillary lymph nodes	Shoulder disarticulation, chemotherapy (adjuvant and neo-adjuvant), radiotherapy	Still alive (>1 year)

Angiosarcomas are malignant mesenchymal vasoformative tumors that arise from lymphatic vessels or vascular endothelial cells and develop in different soft tissues and organs [2-5]. Epithelioid angiosarcoma is a variant of angiosarcoma, with CD31 being the most sensitive and specific marker for endothelial differentiation [17]. The exact mechanisms behind the development of angiosarcoma remain unclear. However, many risk factors, including chronic lymphedema, prior radiation exposure, environmental carcinogens (such as vinyl chloride, thorium dioxide, and arsenic), and certain genetic syndromes, were proposed in the literature and subsequently proven [18]. Moreover, patients on dialysis using an AVF can have an alteration in lymphoid dynamics around the AVF, which can mimic chronic lymphedema. This may lead to a localized immunosuppressed environment [11].

The creation of AVF and the changes in blood flow can result in hypoxia, ischemia, and wall shear stress, all of which are strong proinflammatory triggers that stimulate the release of cytokines such as hypoxia-inducible factor 1 alpha (HIF-1α), immediate-early responsive gene (IEX-1), vascular endothelial growth factor (VEGF), interleukin-1 beta (IL-1β), tumor necrosis factor-alpha (TNF-α), and interferon-gamma (IF-γ) from endothelial cells, leading to inflammation, angiogenesis, and cellular proliferation [16,19]. Furthermore, these factors act as both proliferation stimuli and leukocyte attractants, ultimately leading to intimal hyperplasia, blood flow-related complications of AVFs, and atherosclerosis. Additionally, immunosuppression is a significant risk factor for the development of malignancies (including transplant recipients or patients on immunosuppressive therapy for other reasons) [19]. Pain and swelling or a growing lesion/mass at the fistula site were the most frequent symptoms in the reported cases. These symptoms are nonspecific. Aneurysmal dilation, thrombosis, or localized infection of an AVF can mimic angiosarcoma but are more common than angiosarcoma. Yet, physicians should keep in mind the possibility of angiosarcoma, as early detection is crucial due to the poor prognosis and high mortality rate associated with metastatic disease [11,12].

Diagnostic imaging (such as ultrasound, CT, and MRI) plays an important role in the initial diagnosis, but the definitive diagnosis is based on histological and immunohistochemical examinations. Therefore, every time an AVF is excised, sending the specimen for histology is vital [13]. Furthermore, the ideal primary treatment for non-metastatic disease is radical surgery with complete resection (negative margins) if feasible along with perioperative RT. RT combined with paclitaxel has been shown to achieve durable responses in patients with cutaneous angiosarcoma. In metastatic disease, chemotherapy (such as paclitaxel) is considered the first-line treatment. Targeted therapies and immune checkpoint inhibitors are currently being explored as potential options for the treatment of angiosarcoma [20].

## Conclusions

Angiosarcoma is a highly malignant tumor with a high mortality rate, especially if not detected early. Epithelioid angiosarcoma arising from an AVF is rare, and many common differential diagnoses can mimic it. Due to its nonspecific signs and symptoms, it is often mistaken for more common complications like thrombosis or localized infection. A histopathological examination is critical for establishing an accurate and definitive diagnosis; hence, it is essential with every excisional procedure of an AVF. Timely and precise diagnosis, along with appropriate intervention, are essential to improving patient outcomes even for this aggressive and challenging tumor.

## References

[1] Costache M, Ene AM, Simionescu O, Sajin M (2010). Histopathological diagnosis of cutaneous vascular sarcomas. Rom J Morphol Embryol.

[2] Mobini N (2009). Cutaneous epithelioid angiosarcoma: a neoplasm with potential pitfalls in diagnosis. J Cutan Pathol.

[3] Lucas DR (2009). Angiosarcoma, radiation-associated angiosarcoma, and atypical vascular lesion. Arch Pathol Lab Med.

[4] Rouhani P, Fletcher CD, Devesa SS, Toro JR (2008). Cutaneous soft tissue sarcoma incidence patterns in the U.S.: an analysis of 12,114 cases. Cancer.

[5] Kempson RL (2001). Tumors of the soft tissues. Atlas of Tumor Pathology. Third series, Fascicle 30.

[6] Lucas Álvarez C, Garcia-Cosmes P, Fraile P, Del Carmen Martínez S, Tabernero JM (2013). Angiosarcoma in vascular access after transplantation. Clin Kidney J.

[7] Maddox JC, Evans HL (1981). Angiosarcoma of skin and soft tissue: a study of forty-four cases. Cancer.

[8] Hodkingson DJ, Soule EH, Woods JE (1979). Cutaneous angiosarcoma of the head and neck. Cancer.

[9] Albores-Saavedra J, Schwartz AM, Henson DE, Hruban RH, Goodman ZD, Grayson W, Vollmer RT (2011). Cutaneous angiosarcoma: analysis of 434 cases from the Surveillance, Epidemiology, and End Results Program, 1973-2007. Ann Diagn Pathol.

[10] Buehler D, Rice SR, Moody JS (2014). Angiosarcoma outcomes and prognostic factors: a 25-year single institution experience. Am J Clin Oncol.

[11] Webster P, Wujanto L, Fisher C (2011). Malignancies confined to disused arteriovenous fistulae in renal transplant patients: an important differential diagnosis. Am J Nephrol.

[12] Oskrochi Y, Razi K, Stebbing J, Crane J (2016). Angiosarcoma and dialysis-related arteriovenous fistulae: a comprehensive review. Eur J Vasc Endovasc Surg.

[13] Van Acker P, Bernaert P, Vanrenterghem K, Dierickx D, Reynders D (2020). Angiosarcoma in an arteriovenous fistula after kidney transplantation: case report and review of treatment options. Hemodial Int.

[14] Harrell M, Broehm CJ (2020). Epithelioid variant of angiosarcoma associated with an arteriovenous fistula in the setting of post-renal transplant. Am J Clin Pathol.

[15] Kittitirapong N, Dolmatch BL, Kabutey NK (2021). Angiosarcoma in arteriovenous fistula after kidney transplantation. J Vasc Surg Cases Innov Tech.

[16] D'Ambrosio V, Silvestri P, Aureli F, Sturniolo A, Grandaliano G, Ferraro PM (2020). Angiosarcoma of an arteriovenous fistula for hemodialysis in a kidney transplant recipient affected by Lowe’s syndrome. Case Rep Nephrol.

[17] Ohsawa M, Naka N, Tomita Y, Kawamori D, Kanno H, Aozasa K (1995). Use of immunohistochemical procedures in diagnosing angiosarcoma: evaluation of 98 cases. Cancer.

[18] Florou V, Wilky BA (2018). Current and future directions for angiosarcoma therapy. Curr Treat Options Oncol.

[19] Fitts MK, Pike DB, Anderson K, Shiu YT (2014). Hemodynamic shear stress and endothelial dysfunction in hemodialysis access. Open Urol Nephrol J.

[20] Spiker AM, Mangla A, Ramsey ML (2023). Angiosarcoma. StatPearls [Internet].

